# A Mechanistic Insight into the Anti-Staphylococcal Mode of Action of (+)-Usnic Acid and Its Synergy with Norfloxacin Against Methicillin-Resistant *Staphylococcus aureus*

**DOI:** 10.3390/biom15060750

**Published:** 2025-05-22

**Authors:** Bhavana Gangwar, Santosh Kumar, Parmanand Kumar, Anirban Pal, Mahendra P. Darokar

**Affiliations:** 1 Bioprospection and Product Development Division, CSIR-Central Institute of Medicinal and Aromatic Plants, Lucknow 226015, Indiakumar.santosh@mcm.edu (S.K.);; 2 School of Pharmacy, University of Wisconsin-Madison, Madison, WI 53705, USA; 3 Department of Biological Sciences, The University of Texas at Dallas, Richardson, TX 75080, USA

**Keywords:** antimicrobial resistance, natural therapeutics, gel-free proteomics, gene expression, methicillin-resistant *Staphylococcus aureus* (MRSA), synergy, Usnic acid

## Abstract

In this study, a global response analysis was performed to explore the mechanism of action of Usnic acid and its synergy with Norfloxacin, a well-known quinolone antibiotic to which MRSA clinical isolates showed resistance (MIC, 500 µg/mL). A microdilution assay, a growth kinetics analysis, a microscopic analysis, and cell-based assays consistently showed that Usnic acid possesses strong anti-staphylococcal activity (MIC, 7.8 µg/mL), causes cell leakage, modulates efflux pump activity, and synergizes with Norfloxacin against the multi-drug-resistant clinical isolate MRSA 2071. Whole-cell proteome profiling using gel-free proteomics-based nano-LC-ESI-QTOF-MS/MS revealed several proteins whose expression was significantly modulated by Usnic acid and Norfloxacin alone or in combination. Usnic acid downregulated the abundance of RNA polymerase subunits (RpoB and RpoC), carbamoyl phosphate synthase large subunit (PyrAB), chaperone (GroEL), and adenylosuccinate synthetase (PurA). Interestingly, proteins found to be upregulated in the presence of Usnic acid and Norfloxacin included oxidative-stress-related proteins such as peroxidase (Tpx), alkyl hydroperoxide reductase (AphC), and general stress protein (UspA). This study clearly shows that Usnic acid affects numerous cellular targets and can potentiate the action of Norfloxacin. Furthermore, an in vivo study showed that UA at low concentrations prevents body weight gain, but changes in other tested toxicological parameters were found to be within normal limits. Thus, UA at low doses appears to be a promising candidate for repurposing old antibiotics through combination therapy against MRSA infections.

## 1. Introduction

*Staphylococcus aureus* is a commensal and opportunistic pathogen that extensively contributes to hospital-acquired infections worldwide. The WHO has listed it on the class-2 priority list of pathogens and grouped it with serious bacterial pathogens known as ESKAPE [1]. The growing challenge posed by multi-drug-resistant bacteria, such as methicillin-resistant *Staphylococcus aureus* (MRSA), demands new agents or alternative therapies (such as combination). It is becoming increasingly difficult to develop new antibiotics, and the emergence of resistance to last-resort antibiotics calls for urgent attention to tackle methicillin-resistant *S. aureus* (MRSA) strains [2]. Combination therapy has received much attention in the effort to resensitize bacteria resistant to traditional or clinically used antibiotics [2,3,4].

Multiple resistance mechanisms are involved in the activity of drug-resistant *S. aureus* [5]. As a first line of defense mechanisms, the *S. aureus* genome encodes several transporters that efflux noxious compounds to detoxify cells [6]. Major facilitator superfamily (MFS) proteins such as NorA, NorB, and NorC contribute extensively to conferring resistance to fluoroquinolone (e.g., to Norfloxacin) and dyes such as ethidium bromide in *S. aureus* [6]. The other transporter proteins that are involved in NOR resistance are MdeA, SdrM of the MFS family, and MepA of the toxic compound extrusion (MATE) family; however, their specificity to fluoroquinolones is weak [6,7]. Other mechanisms contributing to drug resistance in *S. aureus* include the enzymatic inactivation of antibiotics (e.g., penicillinase), target alteration (e.g., resistance to methicillin via the expression of a novel penicillin-binding protein, PBP-2a, a product of the *mecA* gene), and mutations in cellular targets (e.g., mutant topoisomerase and DNA gyrase for fluoroquinolone resistance and the *rpoB* gene for rifampin resistance) [5,8].

Natural molecules with antimicrobial activity and synergistic properties can be promising alternatives that can help repurpose old drugs that have become ineffective due to the gain of drug-resistant mechanisms in bacteria. Usnic acid (UA) is a secondary metabolite found in *Lichens* spp. that is present in two natural enantiomers, (+)- or (−)-, and it exerts antimicrobial, antibiofilm, and antiviral activities as well as anti-cancer activity [9,10,11,12,13,14,15]. Both enantiomers of UA can present different biological activities and toxicity in different organisms [14,16]. Its similarities and differences have been reviewed in detail by Galanty et al. (2019) [10]. Compared to (−)-UA, the abundance of (+)-UA and its chemical properties make it more attractive for commercial availability [17]. The antimicrobial activity of both enantiomers against unicellular organisms suggests that both enantiomers possess strong antibacterial activity. Lauterwein et al. found no significant differences in the antibacterial activity of both enantiomers against five out of ten tested bacteria strains, including *S. aureus* [18]. In an interesting study by Lage et al. [19], it was found that both enantiomers of UA inhibited the growth of six clinical isolates of *Helicobacter pylori*, and (+)-UA was at least two-fold more active than (−)-UA.

A previous study on (−)-UA conducted in our lab indicated that UA can damage membranes [15] and downregulate peptidoglycan/fatty acid biosynthesis [20] in MRSA. Some more possible mechanisms of the antibacterial action of UA have been suggested in previous studies. UA was shown to inhibit RNA, DNA, and protein synthesis in *B. subtilis* and *S. aureus* [12]; bacterial membrane damage in MRSA strains [15]; and biofilm inhibition in various bacteria [13]. UA was shown to cause the dissipation of membrane potential in isolated liver mitochondria and bacterial cells [21]. In another study, UA inhibited biofilm development by Gram-positive bacteria through, for example, intra- and interspecies signaling mechanisms based on quorum sensing and cell density [22]. It was also suggested that UA exerts antimicrobial and antibiofilm activity against vancomycin-resistant enterococcus [23] as well as *S. aureus* through the inhibition of peptidoglycan and impaired matrix protein [24].

UA has also shown synergistic potential and has been demonstrated to be a good candidate for the development of new antimicrobial agents or combinations of drugs for chemotherapy [25]. The synergistic effects of UA with colistin against colistin-resistant *Enterobacteriaceae* were reported recently by Zhang et al. in 2023 [26]. UA also showed an antimycobacterial effect against drug-sensitive and drug-resistant tuberculosis [27]. The synergy of UA and Polymyxin against clinical isolates of *Pseudomonas aeruginosa* was reported by da Costa et al. [28]. An evaluation of UA derivatives’ antimicrobial activity against various bacteria and fungi suggested antimicrobial potential based on the strain background [29]. (+)-Usnic acid induced mitochondria-derived reactive oxygen species (ROS) production in lung squamous cell carcinoma cells, causing apoptosis by inhibiting mitochondria respiratory chain complexes and Nrf2 expression [30].

However, the molecular mechanism of antibacterial action requires further investigation. Hence, more studies are required to decipher the underlying molecular mechanisms of antibacterial and synergistic activity to find precise targets to develop alternative therapeutics. An increasing number of herbal and dietary supplement-induced hepatotoxicities have been reported worldwide, and the US Food and Drug Administration (FDA) has raised concerns over its use in dietary supplements and ordered the recall of all products containing UA [31]. Some studies have suggested that UA can be a safe and stable agent for topical uses [32]. Attempts to reduce toxicity and increase efficacy were made by different approaches, such as chemical modifications and encapsulated delivery, e.g., nanoencapsulation [33]. Besides its documented antimicrobial activity, UA should be used with extreme caution to combat drug-resistant pathogens.

In this work, different cell-based assays, a gene expression analysis, and a whole-cell proteome analysis were performed to delineate the molecular mechanism of anti-staphylococcal activity of (+)-UA (referred to as UA) and the synergy mechanism with NOR. The high-throughput and sensitive approach based on gel-free whole-cell proteome analysis by nan-LC-ESI-QTOF helped us analyze and identify a broad range of proteins, including less abundant ones (expressed in small quantities) that are hard to detect by gel-based approaches such as 2D-PAGE. To the best of our knowledge, this is the first systematic report describing the molecular basis of the synergy of UA with antibiotic NOR against MRSA. In addition, an in vivo study was undertaken to assess the toxicity and efficacy of UA in combination with NOR using the Swiss albino mice model.

## 2. Materials and Methods

### 2.1. Bacterial Strains, Culture Conditions, Chemicals, and Enzymes

*S. aureus* reference strain MTCC 96 (ATCC 9144) and MRSA clinical isolates [15,34] were maintained and cultured in standard Mueller–Hinton agar and cation-adjusted Mueller–Hinton broth media (MHA and CAMHB, Hi-Media, Mumbai, India). (+)-Usnic acid (UA), Norfloxacin (NOR), Oxacillin (OXA), Cefoxitin (FOX), Cefazolin (CFZ), Ciprofloxacin (CIP), Tetracycline (TET), Erythromycin (ERY), Streptomycin (STR), Vancomycin (VAN), iodonitrotetrazolium chloride, phosphate-buffered saline, dimethyl sulfoxide (DMSO), thiourea, and lysostaphin were procured from Sigma-Aldrich (St. Louis, MO, USA). UA stock solution (2 mg/mL) was prepared in DMSO. Trypsin Gold, mass spectrometry grade, was purchased from Promega (Madison, WI, USA). Taq DNA polymerase, a cDNA synthesis kit, and SYBR GreenER qPCR SuperMix were purchased from Invitrogen (Carlsbad, CA, USA).

### 2.2. Determination of Minimum Inhibitory Concentrations (MICs) 

MIC values of UA and clinically used antibiotics (of various classes) were evaluated against MRSA clinical isolates (Table S1) using the broth microdilution assay as per the CLSI guidelines 2018 [35]. DMSO was used as a control. *S. aureus* MTCC 96 (ATCC 9144) was used as a reference strain.

### 2.3. In Vitro Synergy of UA with Different Antibiotics

The Checkerboard method was used as described previously [36]. Briefly, UA was tested in combination with various groups of antibiotics including beta-lactams (Oxacillin, Cefoxitin, and Cefazolin), fluoroquinolone (Norfloxacin and Ciprofloxacin), protein synthesis inhibitors (Tetracycline, Erythromycin, and Streptomycin), and glycopeptide (Vancomycin) against MRSA clinical isolates. The assay was performed using sterile 96-well ‘U’-bottom plates (Genaxy, New Delhi, India). Antibiotics ranging from 1000 to 7.8 µg/mL were used in combination with UA (7.8 to 0.03 µg/mL) in a two-fold serial dilution. In addition, each well, except for the negative control, was inoculated with a bacterial culture of 5 × 10^6^ CFU (colony-forming units)/mL (exponential growth phase) and incubated at 37 °C for 24 h. The Fractional Inhibitory Concentration Index (FICI) was calculated as follows: FICI = FIC of drug A + FIC of drug B (FIC_A_ = MIC of drug A in combination/MIC of drug A alone, and FIC_B_ = MIC of drug B in combination/MIC of drug B alone). The FICI was interpreted as follows: ≤0.5 synergy; >0.5–1.0 additive; >1.0 to <4.0 no interaction; and >4.0 antagonism [37].

### 2.4. Growth Kinetics Study of MRSA 2071

Growth kinetics was performed to assess the effect of NOR, UA, and the combination of NOR + UA on a clinical isolate of MRSA 2071 as per the method described previously [34].

### 2.5. Scanning Electron Microscopy (SEM)

MRSA 2071 cells were treated with 1/2 MIC of NOR, UA, and a combination of UA + NOR and were cultured mid-exponential growth phase (OD600 ≈ 0.6) at 37 °C with shaking (200 rpm). Samples were prepared as described previously [34]. Samples were gold-coated with a Q150TES sputter coater (Quorum Technology, East Sussex, UK) and observed using a Scanning Electron Microscope (FEI Quanta 250 FEG-SEM, Thermo Scientific, Waltham, MA, USA).

### 2.6. Cytoplasmic Leakage Assay

MRSA 2071 was cultured in 25 mL MHB and incubated at 37 °C for 24 h with shaking (200 rpm). After incubation, the bacterial cells were pelleted by centrifugation at 4000× *g* for 10 min at 4 °C. Cell pellets were washed twice and resuspended in sterile normal 1× PBS. The cell concentration was adjusted to ~10^6^ CFU/mL. Suspensions of bacterial culture were challenged with different concentrations of NOR alone, UA alone, and the combination of NOR + UA (1/2 MIC to 4 MIC, each) except for the control (only cells). Suspensions were shaken for 15, 30, 60, 90, and 120 min at 37 °C. The absorbance of supernatants was measured at 260 nm and 280 nm using a spectrofluorometer (FLUOStar Omega BMG Labtech, Offenburg, Germany) [15].

### 2.7. Ethidium Bromide (EtBr) Efflux Assay 

To identify the role of efflux-mediated drug resistance in MRSA 2071, the fluorometric determination of ethidium bromide (EtBr) efflux was performed as per the method described elsewhere [38]. See Supplementary Materials.

### 2.8. Relative Expression of Efflux Pump Genes

First, the inducibility of NOR (1/2 MIC, 1/4 MIC, 1/8 MIC, and 1/16 MIC) on the transcript (mRNA) levels of efflux pump-related genes *norA, norC, mdeA,* and *abcA* was analyzed in cells grown in the mid-exponential growth phase (OD600 ≈ 0.6) at 37 °C with shaking (200 rpm). The relative expression (RQ) of target genes of interest was analyzed using ^∆∆^Ct values compared to the untreated control as described earlier [34]. The effect of UA and the combination of UA + NOR on the gene expression of efflux transporters was tested under similar conditions.

### 2.9. Proteome Profiling for Identification of Differentially Expressed Protein by Nano-LC-ESI-QTOF-MS/MS

Protein extraction was performed on the cells grown (MRSA 2071) with and without treatments with NOR (1/4 MIC of NOR alone), UA (1/4 MIC of UA alone), and a combination of UA + NOR (1/4 MIC of combination) in 25 mL flasks in the mid-exponential growth phase (OD600 ≈ 0.6) at 37 °C with shaking (200 rpm) (extraction method is provided in Supplementary Materials) [39]. Trypsin digestion and protein identification were performed as described earlier [34,40].

### 2.10. Validation of Identified Protein Through RT-qPCR

Expression levels of the differentially expressed proteins found in MRSA 2071 exposed to NOR (1/4 MIC), UA (1/4 MIC), and NOR + UA (1/4 MIC of combination) were determined using gene-specific primers as described previously [34].

### 2.11. Effect of Thiourea on Survival of MRSA 2071

Pre-grown overnight cells were inoculated (~10^6^ CFU/mL) in fresh sterile MHB media (with and without thiourea added), and a set of cultures were challenged with different concentrations of UA, NOR, and UA + NOR (1/4 MIC to MIC each). The cultures were incubated for 12 h at 37 °C with shaking, and optical density (OD_600nm_) was measured every 1 h with a 96-well microplate reader (Thermo Fisher Scientific, Waltham, MA, USA). Each test was carried out in triplicate, averaged, and graphed using OD_600nm_ on the Y-axis and time on the X-axis [34].

### 2.12. Determining In Vivo Efficacy Using Swiss Albino Mice Model

UA alone (2 mg/kg body weight), NOR alone (5 mg/kg body weight), and the combination of NOR + UA (5 + 2 mg/kg body weight) were tested, and a positive control (vancomycin 1 mg/kg body weight) and a negative control (vehicle) were included (n = 5). The infection was accomplished through the intra-venous (i.v.) route with 1 × 10^6^ CFU/mL of *S. aureus* (MTCC 96). The mice were sacrificed after 7 days of therapy to determine the microbial load per gram of spleen and liver tissue using the plate dilution method described by Gupta et al., 2012 [15].

### 2.13. In Vivo Toxicity Assessment of UA 

The Organisation for Economic Co-operation and Development (OECD)’s test guideline No. 423 was followed to evaluate in vivo toxicity in Swiss albino mice [15]. Doses of UA, NOR, and a combination of NOR + UA were administered as above. Blood was collected for various physiological and biochemical markers after the animals were examined for body weight changes. The animals were sacrificed to perform large organ necropsy and the calculation of relative organ weights. Biochemical changes were examined using the method described by Gupta et al., 2012 [15].

### 2.14. Statistical Analysis

Statistical analyses were conducted using Microsoft Excel 365 and GraphPad Prism 5 software. The controls and test data were acquired in triplicate and presented as mean ± standard deviation. Statistical analysis was performed using two-way ANOVA (ns, *p* > 0.05, * *p* < 0.05, ** *p* < 0.01, *** *p* < 0.001; control vs. treatment). 

## 3. Results

### 3.1. The Anti-Staphylococcal Activity of UA and Its Synergy with Antibiotics

MIC of UA was found to be 7.8 µg/mL against MRSA clinical isolates and 3.9 µg/mL against a drug-sensitive strain, MTCC 96 (Table 1). In a combination study, UA in combination with NOR (NOR + UA) showed a synergistic interaction against all MRSA clinical isolates and reduced the MIC by 4-fold. (Table 1). UA reduced the MICs of Oxacillin, Cefoxitin, Cefazolin, Ciprofloxacin, Tetracycline, Erythromycin, and Streptomycin by 2–4-fold and Vancomycin by 4–8-fold (Table 2). The Fractional Inhibitory Concentration Index (FICI) was calculated to identify whether the interactions of UA with the various tested antibiotics are synergistic or additive (Table 2). It was found that the combination of NOR + UA showed synergy against all MRSA clinical isolates; therefore, the combination of NOR + UA was considered for further study.

### 3.2. Growth Kinetics Study 

**Figure 1:**
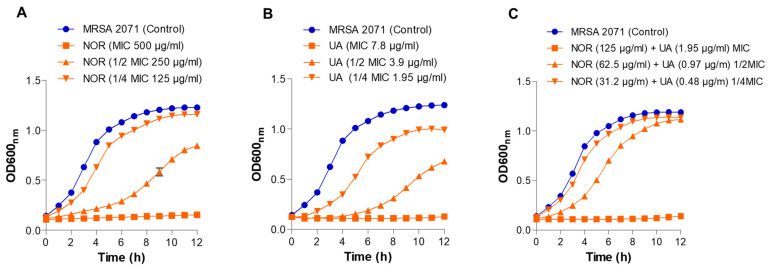
Growth kinetics study of MRSA 2071 under control condition and treated with different concentrations of (**A**) Norfloxacin (NOR; MIC 500, 1/2 MIC 250, and 1/4 MIC 125 µg/mL), (**B**) Usnic acid (UA; MIC 7.8, 1/2 MIC 3.9, and 1/4 MIC 1.95 µg/mL), and (**C**) Norfloxacin + Usnic acid (combination) (combination of NOR + UA MIC, NOR 125 + UA 1.95 µg/mL, 1/2 MIC of combination, NOR 62.5 + UA 0.97 µg/mL and 1/4 MIC of combination, and NOR 31.2 + UA 0.48 µg/mL). Data represent average value obtained from three independent experiments. Graph represents average value from three independent assays, and error bar shows standard deviation (±SD).

Growth kinetics showed no growth of MRSA 2071 at the MIC levels of both UA and NOR (Figure 1). Compared to the untreated cells, treatments with sublethal doses of both UA and NOR partially inhibited growth and were found to increase the lag period of MRSA 2071 in a dose-dependent manner (1/2 MIC > 1/4 MIC). However, the combination of UA + NOR (at 1/4 MIC levels of individual) resulted in complete growth inhibition as effectively as the individual MIC levels of either UA or NOR (Figure 1). Thus, the growth kinetics observations were also found to be consistent with the microdilution assay where UA + NOR (1/4 MIC of each in combination) synergize to arrest the growth of MRSA 2071 (Figure 1).

### 3.3. Conducting a Cell Morphology Study by Scanning Electron Microscopy

**Figure 2:**
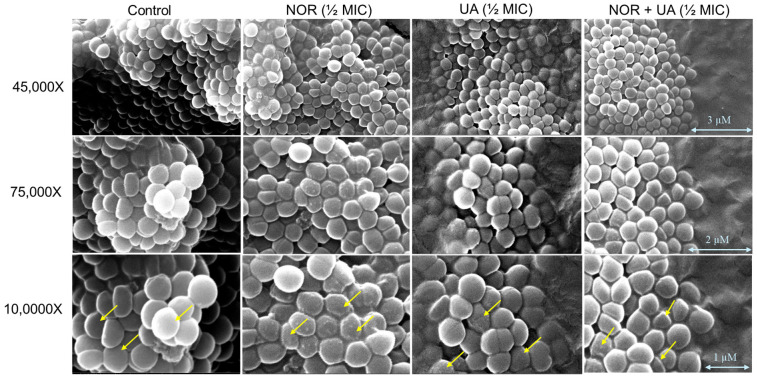
A Scanning Electron Microscopy (SEM) analysis of MRSA 2071 treated with Norfloxacin, Usnic acid, and Norfloxacin + Usnic acid. Exposure to Norfloxacin (1/2 MIC 250 μg/mL) and, to a lesser extent, with Usnic acid (UA, 1/2 MIC 3.9 μg/mL), caused a rough cell morphology compared to the untreated control (indicated by arrows). In a combination of both Norfloxacin and Usnic acid at lower concentrations (NOR + UA 1/2 MIC of combination; NOR 62.5 + UA 0.975 μg/mL) also showed a rough morphology compared to the control.

The UA treatment showed the development of a rough cell morphology; however, the observed effect was more subtle than that of the NOR treatments (Figure 2). The combination of both NOR + UA also showed a rough cell morphology, which indicated a possible membrane damage/cell leakage of cells in the presence of treatments.

### 3.4. Cell Leakage Assay

**Figure 3:**
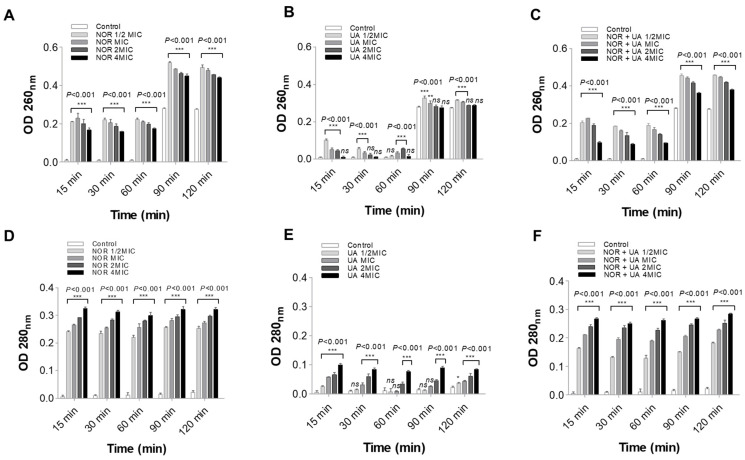
Spectrophotometer-based measurements of cytoplasmic leakage of nucleic acid at 260 nm and proteins at 280 nm in the extracellular medium of MRSA 2071. Leakage shows the relative abundance of protein and Nucleic acid, (**A**,**D**) in the presence of Norfloxacin alone at various concentrations (NOR; 1/2 MIC 250, MIC 500, 2 MIC 1000, and 4 MIC 2000 µg/mL), (**B**,**E**) in the presence of Usnic acid alone (UA; 1/2 MIC 3.9, MIC 7.8, 2 MIC 15.6, and 4 MIC 31.2 µg/mL), (**C**,**F**) in the presence of Norfloxacin and Usnic acid in combination (NOR + UA 1/2 MIC of combination; NOR 62.5 + UA 0.97 µg/mL, MIC of combination; NOR 125 + UA 1.95 µg/mL, 2 MIC of combination; NOR 250 + UA 3.9 µg/mL, 4 MIC of combination; NOR 500 + UA 7.8 µg/mL), respectively. The graph presents the average value from three independent experiments, and the error bars represent the ± SEM and two-way ANOVA results (ns = non-significant; *p* > 0.05, * *p* < 0.05, ** *p* < 0.01, *** *p* < 0.001; control vs. treatment). The experiments were repeated at least twice with n = 3 per experiment.

Cell leakage was assessed by exposing cells to different treatment conditions for a short time. The exposure of MRSA2071 to NOR and UA caused more leaky membranes than the control samples; however, a higher optical density (OD260 for nucleic acid and OD280 for proteins) was observed in the NOR treatment compared to the UA treatment (Figure 3). Interestingly, similar to NOR at high concentrations, the combination of NOR + UA also caused the leakage of cell contents but at lower concentrations, therefore suggesting synergy between the two.

### 3.5. Efflux Pump Assay

**Figure 4:**
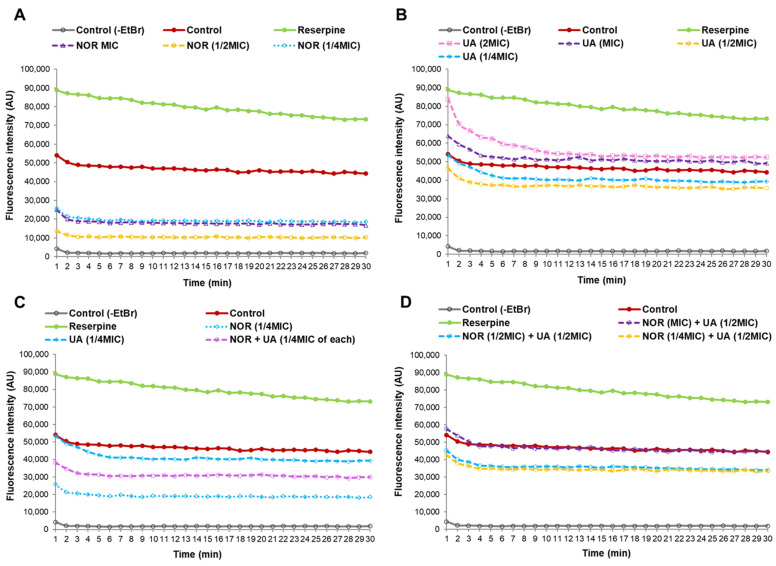
Ethidium bromide assay for efflux pump activity of MRSA 2071 under control conditions and treated with different concentrations of Norfloxacin, Usnic acid, and combination of Norfloxacin + Usnic acid. (**A**) Fluorescence of EtBr in presence of different concentrations of Norfloxacin (NOR; MIC 500, 1/2 MIC 250, and 1/4 MIC 125 µg/mL) in comparison to control. (**B**) Dose-dependent inhibition or activation of efflux activity observed under different concentrations of Usnic acid (UA; 2 MIC 15.6, MIC 7.8, 1/2 MIC 3.9, and 1/4 MIC 1.95 µg/mL), (**C**) Reduced efflux activity in combination of NOR (1/4 MIC of combination, 31.25 µg/mL) + UA (1/4 MIC of combination, 0.48 µg/mL). (**D**) Inhibitory effect of UA (1/2 MIC of combination, 3.9 µg/mL) on NOR-induced efflux activity at various concentrations (1/4 MIC to MIC of NOR in combination).

The EtBr fluorescence measurements found that the treatment of MRSA 2071 with EtBr (+EtBr control) can produce fluorescence compared to the non-treated samples (−EtBr control) (Figure 4A). Reserpine, a well-known efflux pump inhibitor, was used as a positive control, which showed higher fluorescence in comparison to the EtBr control (+EtBr) (Figure 4A). Sublethal concentrations of NOR (1/4 MIC, for 1 h) showed a decrease in fluorescence compared to the control (+EtBr), which suggests that NOR can induce efflux pump activity in MRSA 2071. Further decreased fluorescence or, in other words, enhanced efflux activity (~2 folds) was detected when the concentration of NOR was increased to 1/2 MIC. However, increasing the NOR concentration to MIC and 2 MIC did not increase the efflux activity further, yet it surprisingly slightly reduced the efflux activity as the observed fluorescence increased compared to 1/2 of the MIC of NOR. This suggests that NOR at a sublethal concentration (1/4 MIC and 1/2 MIC) induces an efflux system, but a lethal concentration of NOR (MIC and 2 MIC) damages the activity of efflux pump proteins (Figure 4A). 

UA (1/4 and 1/2 MIC, 1 h) slightly induced efflux activity. However, inducibility in efflux activity due to UA was found to be much lower compared to the NOR treatment (Figure 4B). Importantly, it was observed that an increased concentration of UA (MIC and 2 MIC) strongly inhibited efflux activity (Figure 4B). These findings suggest that UA, above a critical concentration, potentially damages or blocks efflux pump activity. An efflux study of the combination (NOR + UA) clearly showed that UA can decrease the NOR-induced efflux pump activity as higher fluorescence was observed in the cells treated with UA + NOR at 1/4 MIC of the combination (Figure 4C). Furthermore, UA (1/2 MIC) was able to completely neutralize the inducible effect of NOR (MIC) on efflux pump action, as the observed fluorescence was reversed back to the level of the control (Figure 4D). 

### 3.6. Expression Analysis of Efflux Pump Genes and Drug Transporters Through RT-qPCR

**Figure 5:**
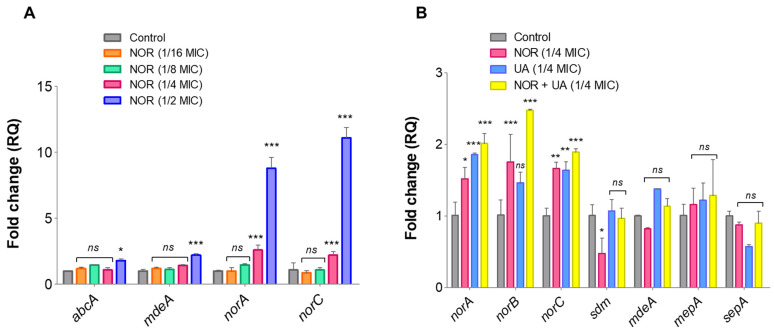
RT-qPCR analysis showing relative expression of genes. (**A**) Relative expression of efflux pump genes in response to different concentrations of Norfloxacin (NOR 1/2 MIC, 250; 1/4 MIC, 125; 1/8 MIC, 62.5; and 1/16 MIC, 31.25 µg/mL) in comparison to control. (**B**) Relative expression of efflux transport genes in response to Usnic acid (UA 1/4 MIC, 1.95 µg/mL) and Norfloxacin + Usnic acid (1/4 MIC of NOR + UA in combination, 31.25 µg/mL + 0.48 µg/mL) in MRSA 2071. Graph presents average value from three independent experiments, and error bars represent ± SEM, two-way ANOVA (ns = non-significant; *p* > 0.05, * *p* < 0.05, ** *p* < 0.01, *** *p* < 0.001).

Compared to the untreated control, the NOR treatment induced the expression of efflux pump genes in a dose-dependent manner (Figure 5A). The effects of UA and NOR + UA were also evaluated on the expression of selected efflux pump genes (Table 3; *norABC*, *sdm*, *mdeA*, *mepA*, and *sepA*). Our findings suggest that, compared to the untreated controls, exposure to a sublethal dose of UA + NOR (1/4 MIC of combination) can induce the expression of efflux pump genes in MRSA 2071 (Figure 5B). Interestingly, the UA treatment (1/4 MIC) also induced the expressions of *norA* and *norC*, but they were less than that with NOR (1/4 MIC) or with the combination (NOR + UA). An increased expression of efflux pump genes at the tested concentration was found in accordance with the EtBr assay, where the addition of a sublethal concentration (1/4 MIC) of NOR and UA induced efflux activity (Figure 4A,B).

### 3.7. Analysis of Differentially Expressed Proteins by Nano-LC-ESI-QTOF-MS/MS 

Treatment using sublethal concentrations (1/4 MIC) of UA and NOR caused the up- and downregulation of several specific and common proteins. Numerous proteins, including metabolic pathways, chaperones, and redox homeostasis-related functions, were identified in MRSA 2071, and a list of all proteins (MS/MS Score > 100) is summarized in Table 4 and Table S2. Exposure to UA significantly downregulated the abundance of various proteins, such as formate acetyltransferase, DNA-directed RNA polymerase subunit beta (RpoB), subunit beta’ (RpoC), translation elongation factors (EF-Tu), translation initiation factors (IF-2 and IF-3), heat shock chaperone (DnaK), glutamine synthetase, and other metabolic enzymes such as enolase, glucosamine-fructose-6-phosphate aminotransferase, glucose-6-phosphate isomerase, and glyceraldehyde-3-phosphate dehydrogenase. Notably, compared to the control, both UA and NOR significantly upregulated proteins related to oxidative stress and cell homeostasis such as peroxidase (Tpx-1), alkyl hydroperoxide reductase (AphC), and general stress protein (UspA). The presence of NOR and NOR + UA increased the abundance of the citric acid cycle and other metabolic pathway-related enzymes, including succinyl-CoA synthetase subunit alpha, pyruvate kinase, isocitrate dehydrogenase, and malate/quinone oxidoreductase. There were also a few common proteins, such as carbamoyl phosphate synthase large subunit, that were significantly downregulated in the presence of both NOR and UA. 

NOR + UA exerted a similar or more intense response than NOR or UA alone, for example, the upregulation of proteins such as malate/quinone oxidoreductase, which again confirms the synergy between the two (Table 4 and Table S2).

Overall, a comparative proteomic analysis suggested that most of the upregulated proteins, in response to UA and NOR, included stress and cell homeostasis-related proteins, while most of the other identified proteins were found to be downregulated in comparison to the control. It is important to note that the individual concentrations of NOR and UA were reduced in combination, and thus, the proteins that were up- or downregulated in response to either NOR alone or UA alone showed a trend reversal in the NOR + UA treatment. However, the proteins that remained up- or downregulated in response to the combination of NOR + UA are likely targeted by the synergistic action of both.

### 3.8. Gene Expression Analysis of Identified Protein Through RT-qPCR

**Figure 6:**
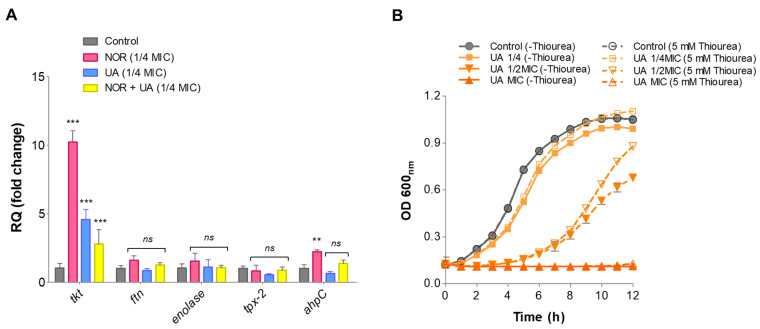
RT-qPCR analysis showing relative expressions of genes identified in proteome analysis of MRSA 2071. (**A**) Relative expression of genes encoding protein biosynthesis and metabolic enzymes and oxidative defense in presence of Norfloxacin (NOR; 1/4 MIC, 125 µg/mL), Usnic acid (UA; 1/4 MIC, 1.95 µg/mL), and combination of NOR + UA (1/4 MIC of NOR in combination = 31.25 µg/mL + 1/4 MIC of UA in combination, 0.48 µg/mL). Graph presents average value from three independent experiments, and error bars represent ± SEM and two-way ANOVA results (ns = non-significant; *p* > 0.05, ** *p* < 0.01, *** *p* < 0.001). (**B**) Growth kinetics of MRSA 2071 grown with or without thiourea and treated with different concentrations of Usnic acid (UA MIC 7.8, 1/2 MIC 3.9, and 1/4 MIC 1.95). Samples with 5 mM of thiourea (scavenger of hydroxyl radicals) showed growth recovery in MRSA 2071 when exposed to sublethal concentration of treatments (dashed curves) compared to control samples lacking thiourea (solid line curves). No thiourea (-thiourea) was used in control to show no inhibition due to 5 mM of thiourea itself in culture medium. Data represent average value obtained from three independent experiments. Graph presents average value from three independent assays, and error bar shows standard deviation (±SD).

The relative gene expression of proteins identified through nano-LC-ESI-QTOF-MS/MS was validated by RT-qPCR under the same treatment conditions. The identified genes involved in oxidative stress defense (*ahpC*, *tpx*, and *ftn*) and a few metabolic enzymes (*eno* and *tkt*) were selected for this analysis (Table 3). Compared to the control, the NOR treatment significantly induced the expression levels of oxidative stress-responsive genes such as *ahpC* (~2.2-fold) and metabolic enzymes such as *tkt* (~10.0-fold) (Figure 6A). NOR + UA (1/4 MIC of combination) also induced the expression of these genes, but to a lower extent than the NOR treatment (Figure 6A). 

### 3.9. Thiourea Rescued Growth Inhibition by UA

Thiourea is a potent hydroxyl radical scavenger, and it was shown to provide protection against antibiotic stress, including the NOR antibiotic [41]. Therefore, to investigate whether thiourea as an antioxidant can provide protection from UA-induced radical stress, the growth of MRSA 2071 was assessed in the presence of various concentrations of thiourea. It was found that the addition of 5 mM of thiourea is non-inhibitory itself but can significantly rescue growth inhibition caused by UA (Figure 6B).

### 3.10. In Vivo Anti-Staphylococcal Efficacy and Toxicity Assessment

The infected, untreated group exhibited a high microbial load, while UA alone (0.5, 01, 05, and 10 mg/kg), NOR alone (01, 05, 10, and 20 mg/kg), and the treatment with the combination of NOR + UA (NOR 05 + UA 01, NOR 10 + UA 01, NOR 05 + UA 02, and NOR 10 + UA 02 mg/kg) significantly (*p* < 0.001) reduced the microbial load in a dose-dependent manner compared to the untreated control (Figure S1). However, the microbial load was found to be slightly higher than that of the standard antibiotic vancomycin, which was used as a positive control. 

To assess the effect of UA on healthy and infected mice, the toxicity studies were separated into two major groups, each with relevant controls. There were no significant changes in body weight in the healthy group that was given an effective dose in a combination of NOR with UA (NOR + UA) continuously for 07 days. There were no significant differences in the oral acute toxicity profile or behavioral, hematological, or serum biochemical markers between the treated and untreated mice (Table 5). However, the gain in body weight was ~38% less in the presence of UA supplemented at a concentration of 2 mg/kg (~5.80 µM) compared to the untreated Swiss albino mice. The inhibition of weight gain was less in the case of UA alone than NOR alone or NOR + UA. Changes in other parameters (shown in Table 5) were found within the normal limits.

## 4. Discussion

This study on (+)-UA demonstrated that it exhibits potent activity (MIC, 3.9–7.8 µg/mL) against clinical isolates (*mecA*^+^) of MRSA that are resistant to several classes of antibiotics, including beta-lactams, fluoroquinolones, and protein biosynthesis inhibitors. Interestingly, UA could re-sensitize MRSA isolates to the different conventional antibiotics in combination (Table 1). The combination of NOR and UA (NOR + UA) showed synergism against all tested MRSA isolates (Table 2). A growth kinetics study also confirmed the synergy between NOR and UA (Figure 1). An SEM analysis of MRSA 2071 in the presence of NOR and UA showed a rough cell morphology (Figure 2). A cell leakage assay showed the presence of proteins and nucleic acids, which suggested damaged cell membranes in MRSA 2071 (Figure 3) [20,41,42,43,44,45,46]. A previous study on (−)-UA by Gupta et al. [15] and further extended work by Sinha et al. [20] found that UA possesses anti-staphylococcal activity in the range of 25–50 µg/mL and showed synergy with antibiotics against clinical isolates used in this study. A variation in the MIC values of both enantiomers could be due to differences in their purity and bacterial strain background. A few studies are available that compare the activity of (+)-UA and (−)-UA. Some studies suggest that both enantiomers of UA possess similar activity against tested bacterial strains [18], but other studies suggest that both enantiomers can have different activities against some other bacterial strains [19]. 

EtBr efflux assays suggest that UA interferes with the efflux pump mechanism, which can re-sensitize MRSA 2071 to NOR [20]. EtBr is a common substrate for many transporters, such as NorA, NorB, NorC, MepA, and SepA in *S. aureus* [47,48]. It was found that NOR, at all tested concentrations, can induce efflux activity (decreased fluorescence) compared to the control (cells +EtBr) (Figure 4A). Interestingly, MIC and 2 MIC of NOR showed reduced efflux activity compared to 1/2 MIC, which suggests that stress caused by NOR at higher concentrations can damage the efflux pump (Figure 4A). UA also modulated efflux action, and its effect varied greatly based on concentration. A sublethal concentration of UA slightly induced efflux activity, but a higher concentration (MIC and 2 MIC) of UA was found to be highly deleterious compared to MIC and 2 MIC of NOR exposure (Figure 4B). The combination of NOR + UA damaged efflux activity more strongly, which further confirms the synergistic activity against MRSA 2071 (Figure 4D). A previous study by Sinha et al. on (−)-UA indicated an inhibitory effect of UA on efflux pump activity, and UA also altered membrane permeability in MRSA [20]. The dose-dependent effect of UA has been documented in other studies where mitochondrial respiration was found to be increased at lower concentrations of UA, but UA showed a destructive effect at higher concentrations due to damage to cell components [49,50,51]. The hepatotoxic effect was found to correlate with oxidative stress caused by UA [52].

The RT-qPCR analysis confirmed that NOR induces the expression of efflux pump genes, specifically *norA* and *norC.* The expression of these genes has been implicated in fluoroquinolones resistance in *S. aureus* [53,54]. UA at lower concentrations also induced the expression of efflux genes *norA,* and *norC* (Figure 5A). Hence, exposure to sublethal concentrations of NOR and UA showed a positive correlation between the observed efflux pump activity and gene expression (mRNA) pattern in MRSA 2071. Both the efflux assay and RT-qPCR experiments suggest that NOR can induce efflux activity in a dose-dependent manner and UA could damage efflux pump activity at higher concentrations (MIC and 2 MIC), and it showed synergy with NOR. 

Furthermore, gel-free proteome profiling was carried out to analyze the global response induced by NOR, UA, and a combination of both. A significant upregulation in various oxidative stress-related proteins, such as peroxidase, alkyl-hydroperoxidase, the universal stress protein UspA, and ribosomal proteins such as L25 and S2 suggested that ROS accumulation occurs in response to both NOR and UA (Table 4). *S. aureus* and other bacteria express various redox-balancing or ROS scavenging systems to escape from the oxidative threat. Some other upregulated proteins in response to NOR and NOR + UA included TCA cycle enzymes, such as malate/quinone oxidoreductase, succinyl-CoA synthetase subunits (alpha and beta), acetate kinase, and isocitrate dehydrogenase. The upregulation of TCA enzymes under antibiotic stress produces oxidative stress in bacteria [41,55]. Treatment using NOR and NOR + UA also upregulated an iron-scavenging protein, ferritin, that is reported to be induced under oxidative stresses in bacteria [56,57].

In response to the UA and NOR treatments, many of the identified proteins were downregulated. UA potentially downregulated the abundance of some proteins, such as DNA-directed RNA polymerase subunit beta (RpoB), molecular chaperone (GroEL), glyceraldehyde-3-phosphate dehydrogenase (GAPDH), and carbamoyl phosphate synthase large subunit, a stationary stage stress protein (Table 4). DNA-directed RNA polymerase subunits (RpoB and RpoC) are reported to contribute to the development of a methicillin resistance phenotype in *S. aureus* as well as multi-drug resistance in several other deadly pathogens like *Mycobacterium* sp. [58,59,60]. Proteins that were more highly affected by UA than NOR could be specific targets of UA. An earlier study suggested that certain proteins are more likely to become induced or damaged by oxidative stresses [20]. Targeting the expression of RpoBC-like proteins would help avoid compensatory mutations in such genes that have been shown to alleviate resistance in several pathogens. In this study, under the treatment conditions, reduced levels of transcription, translation, and ribosomal proteins might be responsible for the low abundance of other proteins in MRSA 2071. Several common proteins affected by both treatments were also regulated similarly with a combination of NOR + UA, which was likely due to the contribution of oxidative stress caused by both molecules. Many antibiotics are now known to cause oxidative stress, and the overexpression of oxidative response in bacteria likely contributes to the development of resistance towards antibiotics. Hence, the development of novel drugs targeting oxidative stress-related proteins could be an attractive strategy to combat drug resistance development in pathogens. 

**Figure 7:**
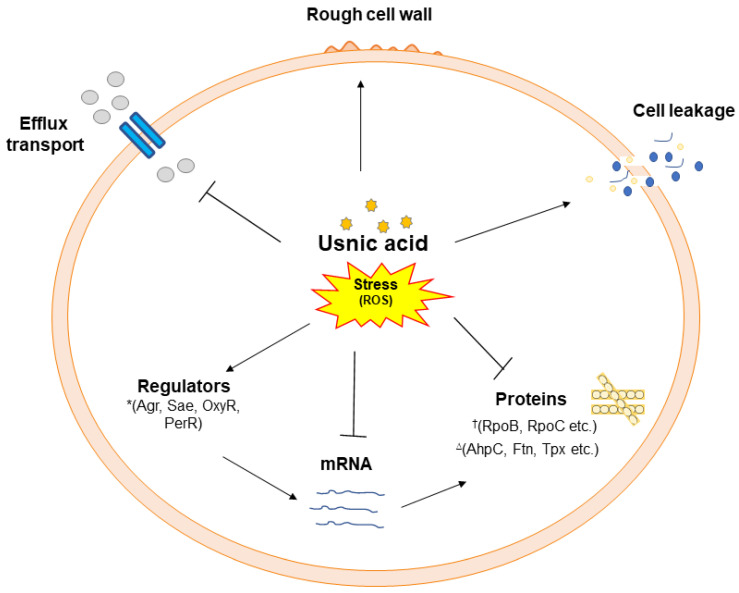
A schematic of Usnic acid’s stress response and the regulation of various targets identified by different cell-based and proteome analyses in MRSA 2071. Usnic acid inhibits efflux transport and downregulates enzymes (such as RNA polymerase subunits RpoB and RpoC) ^†^ and norfloxacin responsive regulators (Agr, Sae, etc.); * Usnic acid induces oxidative responsive proteins (AhpC, Ftn, Tpx, etc.) ^∆^ and cell leakage possibly by enhancing reactive oxygen species such as hydroxyl ions (OH^−^).

The thiourea experiment further confirms that oxidative stress is caused by UA in MRSA 2071 [20,61]. A study by Kohanski et al. 2007 also suggested that antibiotics can produce hydroxyl radicals and that the addition of thiourea could rescue cell damage caused by antibiotic stress [41]. The findings of this study are consistent and suggest that UA targets various cellular components by inducing a stress response that affects the survival of MRSA 2071 (Figure 7). Previous studies on (−)-UA also suggested that oxidative stress is potentially caused by the accumulation of ROS (H_2_O_2_ and NO) in MRSA [20]. Furthermore, the proteome analysis conducted using 2D-PAGE in response to (−)-UA showed the upregulation of several oxidative stress-related proteins [20]. 

Furthermore, in this study, an in vivo toxicity and antibacterial efficacy assessment at low doses of UA and NOR + UA showed promising results. In our experiment, compared to the control, a diet supplemented with UA and NOR prevented weight gain in the Swiss albino mice model, although the other parameters used for the toxicity assessment showed values within the normal limits at the tested concentration (2 mg/Kg body weight) (Table 5). However, to consider UA as safe, more systematic studies are needed to monitor the long-term toxic effects of UA on the physiology of cells. The literature suggests that the FDA documented at least 21 cases of hepatotoxicity by the intake of food supplements containing high doses of UA for slimming purposes (usually ranging from 600 to 1350 mg/day). Earlier studies on the toxicity of UA, in vitro and in vivo, have shown that it can cause serious hepatic reactions, including liver necrosis, fulminant hepatitis, and liver failure. The hepatotoxicity induced by UA is due to its oxidative stress, direct inhibition, and uncoupling of oxidative phosphorylation in liver mitochondria, which decrease ATP levels. The study conducted by Pramyothin et al. in 2004 in isolated rat liver mitochondria suggested that low concentrations of UA (0.15–6 μM) cause uncoupling activity, and high doses (1 mM) of UA can induce the loss of cell membrane integrity by increasing the release of intracellular aspartate aminotransferase (AST) and alanine aminotransferase (ALT) [49]. Furthermore, lipid peroxidation and aniline hydroxylase activity increased, and the glutathione (GSH) content decreased in the cells [49]. Shi et al. demonstrated that UA cytotoxicity (3 and 6 μM after 3 to 20 h) is enhanced by the inhibition of cytochrome P450s (CYP) in primary cultured rat hepatocytes [62]. Several other reports suggest that UA in herbal and dietary supplements like ‘Lipokineti’ causes hepatotoxicity and moderate hepatic injury in normal rats in a dose-dependent manner [63,64]. A study by Han et al. on murine hepatocytes found 98% of cell necrosis, without any sign of apoptosis, 16 h after 5 μM of UA was added to the medium [52]. UA at the tested concentration (5 μM) caused oxidative stress and a significant decrease in the ATP level due to the uncoupling of oxidative phosphorylation and disrupted the normal metabolic pathways [52]. A study by Sonko et al. on rat hepatocytes indicated an increase in oxidative phosphorylation and no changes in cell viability at 1 μM of UA, but 10 μM of UA caused the inhibition of oxidative phosphorylation and decreased cell viability [51]. 

Several in vivo studies have also been carried out to evaluate UA toxicity, which suggested UA toxicity at higher doses. A study by Joseph et al. on mitochondria-specific microarray showed the induction of electron transport chain-related genes only at 600 ppm of UA (=600 mg/L) in mice that were put on a diet with UA at concentrations of 0, 60, 180, and 600 ppm for two weeks. Several other in vivo and in vitro studies that carried out a toxicological assessment for both enantiomers of UA are reviewed elsewhere [31,33]. UA is a promising antimicrobial molecule, but UA intake at higher doses causes toxicity. The topical use of UA was suggested to be safer and well tolerated. Further research is required for the safe consumption of UA and its therapeutic use. It is suggested that UA can be modified or encapsulated to lower the toxicity and its safe delivery while preserving its therapeutic potential for use in animals and humans [65].

## 5. Conclusions

In the present study, it was found that UA exerts strong inhibitory action against MRSA and shows synergy with Norfloxacin. UA causes cell leakage and affects the efflux system in a dose-dependent manner. A gel-free proteome analysis of whole cells found several specific targets of UA that can provide new insights to design and develop novel therapeutics against MRSA. This study revealed several yet unreported targets of UA, such as DNA-directed RNA polymerase subunits (RpoB and RpoC) that contribute to the development of a methicillin-resistant phenotype. Differential protein expression and growth rescue by thiourea suggest that UA affects the redox status in MRSA 2071. Furthermore, an in vivo study in Swiss albino mice suggested that UA is a promising candidate that must be evaluated further in detail through clinical trials for its safety and suitability to be developed as a phytopharmaceutical. In conclusion, using a gel-free proteome analysis, this study not only provides a novel insight into the molecular basis of action of UA and its synergy mechanism with norfloxacin but also provides support for prioritizing a wide range of candidate genes for further study to tackle drug-resistant MRSA.

## Figures and Tables

**Table 1:** Interactions of Usnic acid in combination with antibiotics against clinical isolates of MRSA.

**Clinical Isolates**	**MIC of Antibiotics Alone (µg/mL)**										**MIC of Antibiotics in Presence of Usnic Acid (µg/mL)**								
	**UA**	**OXA**	**FOX**	**CFZ**	**NOR**	**CIP**	**TET**	**ERY**	**STR**	**VAN**	**OXA**	**FOX**	**CFZ**	**NOR**	**CIP**	**TET**	**ERY**	**STR**	**VAN**
MTCC 96	3.9 ± 0.0	0.39 ± 0.11	1.56 ± 0.67	1.56 ± 0.67	0.39 ± 0.45	0.78 ± 0.33	0.78 ± 0.11	0.78 ± 0.33	1.56 ± 0.67	0.78 ± 0.33									
MRSA 2071	7.8 ± 0.0	1000 ± 288.6	500 ± 144.3	500 ± 144.3	500 ± 0.00	500 ± 144.3	25 ± 7.2	1000 ± 288.6	1000 ± 288.6	6.25 ± 1.8	250 ± 72.1	250 ± 72.1	250 ± 72.1	125 ± 0	125 ± 36.0	12.5 ± 7.2	250 ± 72.1	250 ± 0	0.78 ± 0.22
MRSA 4627	3.9 ± 2.2	1000 ± 288.6	500 ± 144.3	500 ± 144.3	500 ± 0.00	500 ± 144.3	50 ± 7.2	1000 ± 288.6	1000 ± 288.6	3.12 ± 5.4	250 ± 72.1	250 ± 72.1	250 ± 72.1	125 ± 0	125 ± 36.0	25 ± 7.2	500 ± 144.3	500 ± 144.3	0.78 ± 0.22
MRSA 1745	7.8 ± 0.0	1000 ± 288.6	500 ± 144.3	500 ± 144.3	500 ± 144.3	500 ± 144.3	50 ± 7.2	1000 ± 288.6	1000 ± 288.6	6.25 ± 1.8	250 ± 72.1	250 ± 72.1	250 ± 72.1	125 ± 36.0	125 ± 36.0	25 ± 7.2	500 ± 144.3	500 ± 144.3	0.78 ± 1.3
MRSA 4423	7.8 ± 0.0	1000 ± 288.6	500 ± 144.3	500 ± 144.3	500 ± 144.3	500 ± 144.3	25 ± 7.2	1000 ± 288.6	1000 ± 288.6	3.12 ± 5.4	250 ± 72.1	250 ± 72.1	250 ± 72.1	125 ± 36.0	125 ± 72.1	12.5 ± 3.6	250 ± 0	250 ± 72.1	0.78 ± 1.3

Abbreviations: UA, Usnic acid; OXA, Oxacillin; FOX, Cefoxitin; CFZ, Cefazolin; NOR, Norfloxacin; CIP, Ciprofloxacin; TET, Tetracycline; ERY, Erythromycin; STR, Streptomycin; VAN, Vancomycin; MIC, Minimum Inhibitory Concentration (µg/mL); ±SD, Standard Deviation.

**Table 2:** Fold reduction in MIC of different antibiotics in combination with Usnic acid, FICI, and interactions between Usnic acid and antibiotics against clinical isolates of MRSA.

Clinical Isolates	OXA		FOX		CFZ		NOR		CIP		TET		ERY		STR		VAN	
	FR (OXA/UA)	FICI/ Interaction	FR (FOX/UA)	FICI/ Interaction	FR (CFZ/UA)	FICI/ Interaction	FR (NOR/UA)	FICI/ Interaction	FR (CIP/UA)	FICI/ Interaction	FR (TET/UA)	FICI/ Interaction	FR (ERY/UA)	FICI/ Interaction	FR (STR/UA)	FICI/ Interaction	FR (VAN/UA)	FICI/ Interaction
MRSA 2071	04/04	0.5/ Synergy	04/02	0.75/ Additive	04/02	0.75/ Additive	04/04	0.5/ Synergy	04/04	0.5/ Synergy	02/02	01/ Additive	04/04	0.5/ Synergy	04/04	0.5/ Synergy	08/08	0.24/ Synergy
MRSA 4627	04/04	0.5/ Synergy	04/02	0.75/ Additive	04/02	0.75/ Additive	04/04	0.5/ Synergy	04/04	0.5/ Synergy	02/02	01/ Additive	02/04	0.75/ Additive	02/04	0.75/ Additive	04/08	0.37/ Synergy
MRSA 1745	04/02	0.75/ Additive	02/04	0.75/ Additive	02/04	0.75/ Additive	04/04	0.5/ Synergy	04/02	0.75/ Additive	02/04	0.75/ Additive	02/04	0.75/ Additive	02/04	0.75/ Additive	08/08	0.25/ Synergy
MRSA 4423	04/04	0.5/ Synergy	02/04	0.75/ Additive	02/04	0.75/ Additive	04/04	0.5/ Synergy	04/02	0.75/ Additive	02/04	0.75/ Additive	04/04	0.5/ Synergy	04/04	0.5/ Synergy	04/08	0.37/ Synergy

Abbreviations: OXA, Oxacillin; FOX, Cefoxitin; CFZ, Cefazolin; NOR, Norfloxacin; CIP, Ciprofloxacin; TET, Tetracycline; ERY, Erythromycin; STR, Streptomycin; VAN, Vancomycin; FR, Fold Reduction; FICI; Fractional Inhibitory Concentration Index; Synergy, when FICI ≤ 0.5; Additive, when 0.5 < FICI ≤ 1.0; Antagonism, when FICI > 4.0.

**Table 3:** List of primers of genes used in this study along with sequences (*S. aureus* N315).

Gene Name	Primer Sequence (5′-3′)
Primers of antibiotic efflux and drug resistance genes	
*abcA*	F-TGCGTCGCCACTTAGATAAC R-GTGGCGACGCAAAATTGGAT
*mdeA*	F-GTTTATGCGATTCGAATGGTTGGT R-AATTAATGCAGCTGTTCCGATAGA
*norA*	F-GGCGGTATATTTGGGGCACT R-TGTCGAGTTCAATCCGCCTG
*norB*	F-ATGGAAAAGCCGTCAAGAGA R-AACCAATGATTGTGCAAATAGC
*norC*	F-ATGAATGAAACGTATCGCGG R-GTCTGCACCAAAACTTTGTTGTAAA
*sdm*	F-AAGCGGTCCAATGATACTCG R-CGCGTGATACAAGGTTTTGG
*mepA*	F-TGCTGCTGCTCTGTTCTTTA R-GCGAAGTTTCCATAATGTGC
*sepA*	F-CCATGATGACCCAAAAATCGA R-GGCGCGACTTTTCATTTG
Primers used for RT-qPCR to validate genes identified through proteome analysis	
*enolase*	F-GGTGCTACAACGTTCAAAGAATCA R-TTCAAATTTAGGAGCGAAACCACC
*tkt*	F-GGTGCTACAACGTTCAAAGAATCA R-TTCAAATTTAGGAGCGAAACCACC
*ahpC*	F-GGTGCTACAACGTTCAAAGAATCA R-TTCAAATTTAGGAGCGAAACCACC
*tpx-2*	F-ATCAAGACGGAACTGTCATTACAAAT R-CTTCTGTGGTACAAGTAGGTGTATTATCTCT
*ftn*	F-AATCGTACGAAGGATTTGCAAAC R-CTTTTGTCCATGGAAACGTTCTT

**Table 4:** A comparative analysis of proteins (top 20 hits) under treatment and control conditions (without treatment).

S.N.	Control	NOR	UA	NOR + UA	Protein Name	Protein MW	pI	Database Accession #	%AA Coverage	MS/MS Search Score	Fold Change		
	# Spectra	# Spectra	# Spectra	# Spectra		(Da)					NOR	UA	NOR + UA
	Total Intensity	Total Intensity	Total Intensity	Total Intensity									
1	31	30	14	30	formate acetyltransferase	85,316.8	5.31	AIA26805.1	45.2	496.38	−1.03	−2.2	−1.03
	2.94 × 10^7^	1.56 × 10^7^	1.96 × 10^6^	2.19 × 10^7^									
2	759	662	363	692	elongation factor Tu	43,159.9	4.74	AIA27105.1	59.6	370.22	−1.14	−2.09	−1.09
	6.76 × 10^8^	5.13 × 10^8^	2.64 × 10^8^	7.39 × 10^8^									
3	9	5	8	13	elongation factor Tu, partial	8757.6	4.96	KMR26954.1	38.2	32.81	−1.8	−1.12	+1.44
	3.81 × 10^7^	3.60 × 10^6^	1.29 × 10^7^	1.22 × 10^7^									
4	90	79	66	96	elongation factor G	76,926.4	4.8	OWU45296.1	40.4	341.22	−1.13	−1.36	+1.06
	1.31 × 10^8^	5.03 × 10^7^	3.35 × 10^7^	1.00 × 10^8^									
5	42	28	22	28	DNA-directed RNA polymerase subunit beta’	135,976.6	6.53	AKK57709.1	23.8	320.81	−1.5	−1.90	−1.5
	3.20 × 10^7^	6.59 × 10^6^	5.42 × 10^6^	1.46 × 10^7^									
6	31	35	22	38	transketolase	72,222.5	4.97	KMS30787.1	46.5	303.16	+1.12	−1.59	+1.22
	2.74 × 10^7^	1.02 × 10^7^	9.59 × 10^6^	3.25 × 10^7^									
7	17	12	3	12	DNA-directed RNA polymerase subunit beta	133,587.5	4.91	ALK38466.1	25.5	285.11	−1.41	−5.6	−1.41
	3.64 × 10^6^	3.43 × 10^5^	2.45 × 10^5^	1.39 × 10^6^									
8	50	55	35	60	1-pyrroline-5-carboxylate dehydrogenase	57,037.7	4.98	AIA29027.1	32.2	276.79	+1.1	−1.42	+1.2
	7.91 × 10^7^	4.40 × 10^7^	2.81 × 10^7^	8.67 × 10^7^									
9	39	41	32	52	aconitate hydratase	99,196.1	4.83	AIA27839.1	25.5	272.58	+1.05	−1.21	+1.3
	3.00 × 10^7^	1.52 × 10^7^	1.04 × 10^7^	3.06 × 10^7^									
10	31	37	20	26	molecular chaperone DnaK	66,417.2	4.65	AIA28120.1	36	268.51	+1.19	−1.55	−1.19
	5.18 × 10^7^	2.82 × 10^7^	1.06 × 10^7^	3.23 × 10^7^									
11	21	27	15	34	malate/quinone oxidoreductase	56,183.1	6.12	KMS00114.1	40.9	268.46	+1.28	−1.4	+1.61
	3.87 × 10^7^	1.61 × 10^7^	5.47 × 10^6^	3.81 × 10^7^									
12	32	52	33	47	succinyl-CoA synthetase subunit beta	42,283.7	4.91	AIA27731.1	40.2	263.16	+1.62	+1.03	+1.46
	4.67 × 10^7^	4.51 × 10^7^	2.16 × 10^7^	5.46 × 10^7^									
13	12	6	2	5	carbamoyl phosphate synthase large subunit	117,669.9	4.87	KMR30550.1	20.3	262.05	−2.0	−6.0	−2.4
	4.99 × 10^6^	1.08 × 10^6^	6.11 × 10^5^	1.81 × 10^6^									
14	33	31	28	36	ATP F0F1 synthase subunit beta	51,399.4	4.68	AIA28597.1	43.1	259.03	−1.06	−1.17	1.09
	2.06 × 10^7^	6.86 × 10^6^	6.68 × 10^6^	1.09 × 10^7^									
15	26	20	14	20	pyruvate kinase	63,329.4	5.24	AIA28225.1	35.2	258.15	−1.3	−1.85	−1.3
	1.29 × 10^7^	5.26 × 10^6^	2.53 × 10^6^	8.53 × 10^6^									
16	28	35	26	22	branched-chain alpha-keto acid dehydrogenase subunit E2	46,454.6	4.9	AHZ98881.1	50.4	256.66	1.25	−1.07	−1.27
	4.98 × 10^7^	3.75 × 10^7^	2.30 × 10^7^	3.28 × 10^7^									
17	43	36	33	44	fructose-bisphosphate aldolase	33,027.9	5.01	QBC22365.1	56	254.66	−1.19	−1.30	1.02
	5.40 × 10^7^	1.84 × 10^7^	1.66 × 10^7^	3.74 × 10^7^									
18	41	42	54	48	2-oxoisovalerate dehydrogenase	35,303.5	4.62	KMR38463.1	43.3	239.53	+1.04	+1.31	+1.17
	6.31 × 10^7^	3.28 × 10^7^	3.58 × 10^7^	4.01 × 10^7^									
19	37	28	20	29	glutamine synthetase	51,125	5.08	AIA27794.1	44.3	239.26	−1.32	−1.85	−1.27
	3.93 × 10^7^	1.92 × 10^7^	5.97 × 10^6^	2.24 × 10^7^									
20	47	28	21	35	enolase	47,173.1	4.55	AIA27354.1	39.6	235.87	−1.67	−2.23	−1.34
	5.65 × 10^7^	2.94 × 10^7^	1.02 × 10^7^	4.32 × 10^7^									

Proteome profiling of MRSA 2071 in the presence of Norfloxacin (NOR; 1/4 MIC, 125 µg/mL), Usnic acid (UA; 1/4 MIC, 1.95 µg/mL), and a combination of Norfloxacin + Usnic acid (NOR; 1/4 MIC of combination, 31.25 µg/mL + UA; 1/4 MIC of combination, 0.48 µg/mL). The upregulation (+) and downregulation (−) of proteins (fold change) were calculated using spectral counts of the treatment and compared to the untreated control samples as described earlier [16,32]. The color intensity represents the relative abundance of proteins under different treatment and control conditions as observed in an analysis using the Spectrum mill software Agilent MassHunter Qualitative analysis B.07.00 (Agilent, Santa Clara, CA, USA). The intensity of the color (ranging from light yellow to dark red) represents a higher abundance of proteins in the samples. A full list of proteins is provided in Supplementary Table S2. A probability score of *p* < 0.05 was set as the criterion for identification. The total protein was pooled from three replicates in each condition and quantified, and an equal amount of protein was processed for identification.

**Table 5:** In vivo study showing effect of Usnic acid on hematological and biochemical changes.

Parameters	Control	UA (2 mg/kg Body Weight)	NOR (10 mg/kg Body Weight)	NOR + UA (10 + 2 mg/kg Body Weight)
Change in body weight (gm)	7.45 ± 1.06	4.63 ± 1.42	0.82 ± 1.36	0.88 ± 2.08
Relative organ weight (gm); (a) spleen and (b) liver	0.24 ± 0.37 0.40 ± 0.33	0.45 ± 0.44 0.56 ± 0.39	0.21 ± 0.04 0.60 ± 0.12	0.12 ± 0.02 0.29 ± 0.05
RBC count (million/mm^3^) (normal range: 4.7 to 6.1 million cells/mcL)	4.89 ± 0.332	5.34 ± 1.078	5.39 ± 1.015	5.91 ± 0.391
WBC count (thousand/mm^3^) (normal range: 4.5 to 11.0 × 10^9^/L)	6.46 ± 0.55	6.44 ± 0.57	10.45 ± 2.58	6.86 ± 0.31
Hemoglobin (g/dL) (normal range for males: 13.8 to 17.2 g/dL; females: 12.1 to 15.1 g/dL)	11.8 ± 0.99	12.3 ± 1.13	12.4 ± 1.27	12.4 ± 1.20
SGPT (U/L) (normal range: 49 U/L)	17.101 ± 0.006	9.11 ± 0.004	16.43 ± 0.002	16.29 ± 0.009
SGOT (U/L) (normal range: 46 U/L)	41.29 ± 0.005	33.155 ± 0.016	34.9 ± 0.0002	34.9 ± 0.0047
Serum creatinine (mg/dL) (normal range for males: 0.6–1.1 mg/dL; females: 0.5–0.8 mg/dL)	0.797 ± 0.004	0.730 ± 0.007	0.784 ± 0.001	0.919 ± 0.004
Serum ALKP (U/L) (normal range for females: 64–306 U/L; males: 80–306 U/L; children: 180–1200 U/L)	220 ± 0.059	90.5 ± 0.039	220 ± 0.018	176.61 ± 0.003
Serum total cholesterol (mg/dL) (normal range: 150–220 mg/dL)	140.97 ± 0.127	116.58 ± 0.089	130.08 ± 0.049	114.14 ± 0.012
Serum bilirubin (mg/dL) (normal range: 1.2 mg/dL)	12.103 ± 7.105	0.102 ± 0.723	0.184 ± 0.024	0.276 ± 0.046
Serum triglycerides (mg/dL) (normal range for males: 60–165 mg/dL; females: 40–140 mg/dL)	69.59 ± 0.034	84.58 ± 0.013 *	65.91 ± 0.027	86.22 ± 0.040 *

* Significant increase in comparison to control.

## Data Availability

The original contributions presented in this study are included in the article/Supplementary Material; further inquiries can be directed to the corresponding author.

## Supplementary Materials

The following supporting information can be downloaded at https://www.mdpi.com/article/10.3390/biom15060750/s1, Figure S1: In vivo efficacy using Swiss albino mice model; Table S1: Determination of minimum inhibitory concentrations (micro-broth dilution assay); Table S2: Complete list of proteins identified by nano-LC-ESI-QTOF-MS/MS analysis under treatment conditions compared to control (without treatment) in clinical isolate of MRSA 2071.
