# Supplementary material for: A Mechanistic Insight into the Anti-Staphylococcal Mode of Action of (+)-Usnic Acid and Its Synergy with Norfloxacin Against Methicillin-Resistant Staphylococcus aureus
Source: Biomolecules. 2025 May 22;15(6):750. doi: 10.3390/biom15060750 (PMC12190719; doi:10.3390/biom15060750)
Supplement: Supplementary file 1 [file biomolecules-15-00750-s001.zip › biomolecules-3155422-supplementary.pdf]

<u>**Supplementary**</u>

MATERIALS AND METHODS

1. EtBt efflux assay

MRSA2071 cells were cultured and grew until mid-exponential growth phase (OD600 ≈ 0.6) at 37 °C with shaking (200 rpm). The cells were collected by centrifugation at 4,000×g for 5 min and washed with phosphate buffer saline (1X PBS). The resuspended cells were treated with an increasing gradient concentration of NOR, UA, and the combinations of NOR+UA (1/4MIC to 2MIC) and ethidium bromide (25 µg/mL) were added subsequently. For positive control, MRSA 2071 was treated with reserpine (25 µg/mL). Treated cells were incubated for 1 h at 25 °C. The EtBr-loaded bacterial cell suspension was centrifuged for 5 min at 4,000×g, the supernatant was discarded, the pellet was washed with 1X PBS, and the cells were resuspended in fresh 1X PBS. The bacterial suspension was aliquoted (0.2 mL) and placed in a 96-well plate. EtBr released by the cell to the suspension was measured by detecting changes in fluorescence for 30 min with regular intervals of 1 min at excitation and emission wavelengths of 530 nm and 585 nm, respectively (1), using a spectrofluorometer (FLUOStar Omega BMG Labtech, Offenburg, Germany).

2. Proteome profiling for identification of differentially expressed protein by nano-LC-ESI-QTOF

For protein extraction, 5 mL cells of equal $OD_{600\ nm}$ (OD600 ≈ 0.6) were collected from each set grown in triplicates by centrifugation at 4000g for 10 min and the supernatant was discarded. Pellets were washed twice with 1X PBS and resuspended in 1.0 mL lysis buffer containing 25 mM Tris (pH 8.0), 5 mM EDTA, 2 mM PMSF, and 25 µg/mL lysostaphin. Cells were incubated for 30 min at 37 °C and chilled on ice before sonication for 3 min (10 sec pulse with a pause of 10 sec). The cell lysate was kept on ice throughout sonication and centrifuged for 30 min at 14,000×g at 4 °C to remove cell debris. Protein concentrations were measured using the Bradford method described previously (2). Proteins were precipitated with 4 volumes of cold acetone for overnight at -20 ºC. Protein pellets were collected by centrifugation at 16000×g for 10 min and air dried. Pellets were dissolved in a resuspension buffer containing 6 M Urea and 100 mm Tris (pH 8.0). Protein samples (10 µg/ µL) in urea buffer were treated with 200 mM DTT for 1 h and subsequently alkylated with 200 mM iodoacetamide for 1 h. The proteins were diluted 10-fold

to reduce the concentration of urea to 0.6 M before digestion with trypsin (1:50 ratio, trypsin: protein).

Figure S1: Invivo efficacy using Swiss albino mice model.

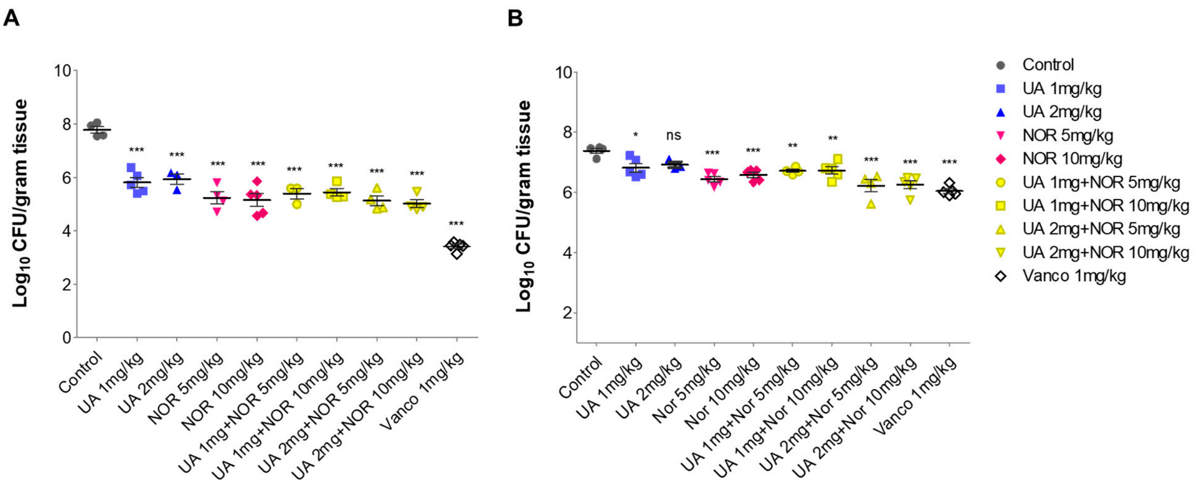

Figure S1: Microbial load determination of **(A)** spleen and **(B)** liver tissue of Swiss albino mice in untreated (control) and treated with different effective doses of NOR (05 mg/kg and 10 mg/kg) and UA (01 mg/kg, 02 mg/kg) as well as in combination of UA + NOR (UA=01 mg/kg+ NOR=05 mg/kg, UA=01 mg/kg+ NOR=10 mg/kg, UA=02 mg/kg+ NOR=05 mg/kg, and UA=02 mg/kg +NOR=10 mg/kg). The bacterial load of tissue was expressed as mean ± SEM, for statistical significance, Dunnett's multiple tests were performed (ns, $P > 0.05$, *$P < 0.05$, **$P < 0.01$, ***$P < 0.001$ control vs treatment)

Table S1. Determination of minimum inhibitory concentrations (Micro-broth dilution assay)

Minimum inhibitory concentrations (µg/mL) of different antibiotics against MRSA clinical isolates through micro-broth dilution assay. As per Clinical and Laboratory Standards Institute guidelines (CLSI-2018).

**Antibiotics**		**Clinical isolates of MRSA**				
		MTCC 96	MRSA 2071	MRSA 4627	MRSA 1745	MRSA 4423
Biomolecule	(+)-Usnic Acid	3.9	7.8	7.8	7.8	7.8
β-lactam group	Oxacillin	3.9	1000	1000	1000	1000
	Penicillin	0.195	1000	500	1000	500
	Carbenicillin	3.9	500	500	500	500
	Ampicillin	0.195	1000	1000	1000	1000

	Cephalosporin	7.8	1000	1000	1000	1000
	Cefazolin	1.56	500	500	500	500
	Cefoxitin	1.56	500	500	500	500
Glycopeptides	Vancomycin	0.78	6.25	3.12	6.25	3.12
	Teicoplanin	0.78	3.12	1.56	3.12	3.12
Lipopeptides	Daptomycin	1.56	3.12	1.56	3.12	1.56
	Colistin	7.8	125	250	250	250
	Bacitracin	7.8	125	31.25	125	125
Aminoglycosides	Amikacin	1.56	250	250	250	250
	Streptomycin	1.56	1000	1000	1000	1000
Macrolides	Erythromycin	0.78	1000	1000	1000	1000
Tetracyclines	Tetracycline	0.78	25	50	50	25
Fluoroquinolones	Ciprofloxacin	0.78	500	500	500	500
	Norfloxacin	0.39	500	500	500	500
	NalidixicAcid	1.56	250	250	250	500
Folate inhibitor	Trimethopim	1.56	1000	1000	1000	1000
Oxazolidinones	Linezolid	1.56	3.12	1.56	3.12	1.56

Values shown in the table are the mean values of three separate experiments performed in triplicates.

Table S2. Complete list of proteins identified by nano-LC-ESI-QTOF analysis under treatment conditions than control (without treatment) in a clinical isolate of MRSA 2071.

S.N.	Control<br># spectra<br>total intensity	NOR<br># spectra<br>total intensity	UA<br># spectra<br>total intensity	NOR +UA<br># spectra<br>total intensity	Protein Name	Protein MW (Da)	pI	Database Accession #	%AA Coverage	MS/MS Search Score	Fold Change		
											NOR	UA	NOR +UA
1	31<br>2.94E+07	30<br>1.56E+07	14<br>1.96E+06	30<br>2.19E+07	formate acetyltransferase	85316.8	5.31	AIA26805.1	45.2	496.38	-1.03	-2.2	-1.03
2	759<br>6.76E+08	662<br>5.13E+08	363<br>2.64E+08	692<br>7.39E+08	elongation factor Tu	43159.9	4.74	AIA27105.1	59.6	370.22	-1.14	-2.09	-1.09
3	9<br>3.81E+07	5<br>3.60E+06	8<br>1.29E+07	13<br>1.22E+07	elongation factor Tu, partial	8757.6	4.96	KMR26954.1	38.2	32.81	-1.8	-1.12	+1.44
4	90<br>1.31E+08	79<br>5.03E+07	66<br>3.35E+07	96<br>1.00E+08	elongation factor G	76926.4	4.8	OWU45296.1	40.4	341.22	-1.13	-1.36	+1.06
5	42<br>3.20E+07	28<br>6.59E+06	22<br>5.42E+06	28<br>1.46E+07	DNA-directed RNA polymerase subunit beta'	135976.6	6.53	AKK57709.1	23.8	320.81	-1.5	-1.90	-1.5
6	31<br>2.74E+07	35<br>1.02E+07	22<br>9.59E+06	38<br>3.25E+07	transketolase	72222.5	4.97	KMS30787.1	46.5	303.16	+1.12	-1.59	+1.22
7	17<br>3.64E+06	12<br>3.43E+05	3<br>2.45E+05	12<br>1.39E+06	DNA-directed RNA polymerase subunit beta	133587.5	4.91	ALK38466.1	25.5	285.11	-1.41	-5.6	-1.41
8	50<br>7.91E+07	55<br>4.40E+07	35<br>2.81E+07	60<br>8.67E+07	1-pyrroline-5-carboxylate dehydrogenase	57037.7	4.98	AIA29027.1	32.2	276.79	+1.1	-1.42	+1.2
9	39<br>3.00E+07	41<br>1.52E+07	32<br>1.04E+07	52<br>3.06E+07	aconitate hydratase	99196.1	4.83	AIA27839.1	25.5	272.58	+1.05	-1.21	+1.3
10	31<br>5.18E+07	37<br>2.82E+07	20<br>1.06E+07	26<br>3.23E+07	molecular chaperone DnaK	66417.2	4.65	AIA28120.1	36	268.51	+1.19	-1.55	-1.19
11	21<br>3.87E+07	27<br>1.61E+07	15<br>5.47E+06	34<br>3.81E+07	malate:quinone oxidoreductase	56183.1	6.12	KMS00114.1	40.9	268.46	+1.28	-1.4	+1.61
12	32	52	33	47		42283.7	4.91	AIA27731.1	40.2	263.16	+1.62	+1.03	+1.46

	4.67E+07	4.51E+07	2.16E+07	5.46E+07	succinyl-CoA synthetase subunit beta								
13	12<br>4.99E+06	6<br>1.08E+06	2<br>6.11E+05	5<br>1.81E+06	carbamoyl phosphate synthase large subunit	117669.9	4.87	KMR30550.1	20.3	262.05	-2.0	-6.0	-2.4
14	33<br>2.06E+07	31<br>6.86E+06	28<br>6.68E+06	36<br>1.09E+07	ATP F0F1 synthase subunit beta	51399.4	4.68	AIA28597.1	43.1	259.03	-1.06	-1.17	1.09
15	26<br>1.29E+07	20<br>5.26E+06	14<br>2.53E+06	20<br>8.53E+06	pyruvate kinase	63329.4	5.24	AIA28225.1	35.2	258.15	-1.3	-1.85	-1.3
16	28<br>4.98E+07	35<br>3.75E+07	26<br>2.30E+07	22<br>3.28E+07	branched-chain alpha-keto acid dehydrogenase subunit E2	46454.6	4.9	AHZ98881.1	50.4	256.66	1.25	-1.07	-1.27
17	43<br>5.40E+07	36<br>1.84E+07	33<br>1.66E+07	44<br>3.74E+07	fructose-bisphosphate aldolase	33027.9	5.01	QBC22365.1	56	254.66	-1.19	-1.30	1.02
18	41<br>6.31E+07	42<br>3.28E+07	54<br>3.58E+07	48<br>4.01E+07	2-oxoisovalerate dehydrogenase	35303.5	4.62	KMR38463.1	43.3	239.53	+1.04	+1.31	+1.17
19	37<br>3.93E+07	28<br>1.92E+07	20<br>5.97E+06	29<br>2.24E+07	glutamine synthetase	51125	5.08	AIA27794.1	44.3	239.26	-1.32	-1.85	-1.27
20	47<br>5.65E+07	28<br>2.94E+07	21<br>1.02E+07	35<br>4.32E+07	enolase	47173.1	4.55	AIA27354.1	39.6	235.87	-1.67	-2.23	-1.34
21	26<br>2.82E+07	25<br>1.37E+07	18<br>6.03E+06	31<br>3.67E+07	ornithine--oxo-acid aminotransferase	43702	5.22	AIA27454.1	40.1	229.65	-1.04	-1.44	+1.19
22	7<br>2.95E+06	0<br>0.00E+00	0<br>0.00E+00	1<br>3.72E+04	ATP-dependent Clp protease ATP-binding subunit	91150.8	5.51	OWU42251.1	20.5	199	-	-	-7.0
23	33<br>3.95E+07	16<br>1.46E+07	17<br>7.11E+06	17<br>1.25E+07	glucosamine--fructose-6-phosphate aminotransferase	65962.4	4.93	AIA28646.1	31.1	213.34	-2.06	-1.94	-1.94
24	61<br>1.22E+08	57<br>6.19E+07	42<br>3.21E+07	60<br>7.26E+07	dihydrolipoamide dehydrogenase	49621.6	4.95	AKK58161.1	32.4	210.84	-1.07	-1.45	-1.01
25	1<br>6.79E+05	0<br>0.00E+00	0<br>0.00E+00	1<br>1.47E+06	ethanol-active dehydrogenase	36446.3	5.35	AIA27170.1	42.8	195.29	-	-	-
26	24<br>2.28E+07	17<br>7.68E+06	14<br>2.37E+06	18<br>1.06E+07	trigger factor	48656.2	4.35	OWU43772.1	36	194.36	-1.4	-1.7	-1.8
27	52	41	37	41	pyruvate dehydrogenase	41381.9	4.9	AIA27583.1	40.5	193.43			

	7.45E+07	2.41E+07	1.60E+07	4.45E+07							-1.2	-1.4	-1.2
28	22<br>5.18E+06	21<br>4.51E+06	16<br>2.56E+06	20<br>9.19E+06	phosphoenolpyruvate--protein phosphotransferase	63416.1	4.66	QBC23603.1	21.6	190.5	-1.0	-1.3	-1.1
29	27<br>2.48E+07	28<br>1.38E+07	13<br>4.00E+06	41<br>2.97E+07	phosphoenolpyruvate carboxykinase (ATP)	59581.4	5.74	OWU39068.1	26.9	187.27	1.0	-2.0	+1.5
30	24<br>2.77E+07	28<br>1.51E+07	19<br>3.93E+06	34<br>3.16E+07	acetate kinase	44098.8	5.65	AIA28238.1	34.7	185.04	+1.1	-1.2	-1.4
31	24<br>8.42E+06	15<br>3.39E+06	13<br>1.67E+06	16<br>2.92E+06	inosine-5-monophosphate dehydrogenase	53021.8	5.54	KMS24688.1	34.6	184.63	-1.6	-1.8	-0.2
32	21<br>2.43E+07	14<br>3.80E+06	19<br>4.26E+06	20<br>1.52E+07	NADP-dependent phosphogluconate dehydrogenase	52001.1	5.02	QBC21287.1	30.7	182.82	-1.4	-1.1	-1.0
33	31<br>3.93E+07	27<br>1.80E+07	15<br>3.02E+05	32<br>3.78E+07	pyridoxal biosynthesis protein	32106.3	5.1	AIA27076.1	42.3	177.95	-1.1	-2.0	1.0
34	12<br>6.33E+06	13<br>2.99E+06	6<br>2.97E+05	20<br>1.21E+07	30S ribosomal protein S1	43286.5	4.51	AIA27961.1	39.3	174.24	0.9	-2.0	1.5
35	18<br>4.81E+06	12<br>3.37E+06	10<br>4.38E+05	15<br>3.68E+06	glutamyl-tRNA synthetase	53712.8	5.21	AIA28420.1	29.6	171.36	-1.5	-1.8	-1.2
36	15<br>4.15E+06	12<br>1.70E+06	6<br>2.17E+05	9<br>2.88E+06	translation initiation factor IF-2	78013.5	5.07	AKK60959.1	21.5	166.34	-1.2	-2.5	-1.6
37	17<br>1.48E+07	16<br>6.00E+06	10<br>4.69E+06	15<br>1.05E+07	glycine dehydrogenase subunit 2	54923.3	5.63	QCV68153.1	26.9	163.21	-1.0	-1.7	-1.1
38	20<br>1.43E+07	25<br>1.40E+07	23<br>7.80E+06	26<br>1.95E+07	isocitrate dehydrogenase	46508.9	4.84	KMS49865.1	31	162.77	1.2	1.1	1.3
39	9<br>1.92E+07	5<br>4.53E+06	8<br>2.83E+06	18<br>9.79E+06	thymidine phosphorylase	46374.3	4.95	AHZ99966.1	27.2	161.85	-1.8	-1.1	1.3
40	25<br>1.16E+07	21<br>4.18E+06	15<br>2.93E+06	25<br>6.80E+06	phosphotransacetylase	35092.2	4.73	KMR52508.1	42.9	159.96	-1.1	-1.6	1.0
41	18<br>1.28E+07	10<br>4.41E+06	10<br>3.39E+06	13<br>7.98E+06	peptigoglycan-binding protein LysM	55949.8	5.58	ALK35437.1	22.8	158.16	-1.8	-1.8	-1.3
42	15<br>1.66E+07	15<br>3.64E+06	6<br>1.06E+06	15<br>5.75E+06	glucose-6-phosphate isomerase	49864.3	4.83	AIA27460.1	25.7	157.85	1.0	-2.5	1.0

43	19<br>1.35E+07	16<br>5.11E+06	11<br>1.52E+06	21<br>1.21E+07	ATP F0F1 synthase subunit alpha	54640.1	4.91	AIA28599.1	28.6	156.91	-1.1	-1.7	1.1
44	29<br>3.43E+07	20<br>9.32E+06	23<br>3.65E+06	24<br>2.04E+07	elongation factor Ts	32607.4	5.05	AIA27743.1	35.4	156.36	-1.4	-1.2	-1.2
45	14<br>5.43E+06	7<br>2.32E+06	6<br>1.72E+06	13<br>4.14E+06	glycerol kinase	55795.7	4.94	AIA27785.1	23.2	151.32	-2.0	-2.3	-0.9
46	12<br>5.76E+06	13<br>4.64E+06	9<br>9.08E+05	17<br>8.09E+06	oxidoreductase	115945.2	5.04	AIA28792.1	10.8	151.31	1.0	-1.3	-1.4
47	28<br>3.66E+07	30<br>1.95E+07	19<br>1.71E+07	21<br>2.02E+07	50S ribosomal protein L5	20266.3	9.32	AIA28725.1	56.4	151.26	1.0	-1.4	-1.3
48	14<br>4.88E+06	10<br>1.42E+06	6<br>8.68E+05	10<br>3.42E+06	cysteine synthase	33032.1	5.39	AIA27070.1	53.8	140.33	-1.4	-2.3	-1.4
49	13<br>7.34E+06	14<br>2.55E+06	11<br>3.00E+06	15<br>4.68E+06	glutamate dehydrogenase	45931.4	5.15	AIA27455.1	28.7	139.9	1.0	-1.1	-1.1
50	11<br>1.24E+07	11<br>7.31E+06	9<br>1.68E+06	14<br>1.58E+07	formate--tetrahydrofolate ligase	60081.7	5.77	KMS19158.1	19.8	139.41	1.0	-1.2	1.2
51	7<br>8.72E+06	8<br>6.09E+06	7<br>1.68E+06	9<br>1.17E+07	formate--tetrahydrofolate ligase	60061.6		AHZ99579.1	20	135.79	1.1	1.0	1.2
52	17<br>9.16E+06	14<br>5.67E+06	9<br>1.76E+06	14<br>7.00E+06	glycine cleavage system protein T	40628.3	4.8	AIA28077.1	29.2	138.74	-1.2	-1.8	-1.2
53	23<br>3.00E+07	22<br>1.47E+07	21<br>1.34E+07	35<br>3.61E+07	fructose-bisphosphate aldolase	30949.6	5.01	AIA28619.1	33.5	138.25	1.0	1.0	+1.5
54	360<br>4.77E+08	290<br>2.73E+08	200<br>1.85E+08	330<br>5.01E+08	DNA-binding protein	9625.8	9.52	AIA27958.1	54.4	136.4	-1.2	-1.8	-1.0
55	15<br>3.97E+06	13<br>1.97E+06	6<br>1.05E+06	15<br>2.31E+06	glutamyl-tRNA synthetase	56344.8	5.21	AKK57694.1	22.9	136.26	-1.1	-2.5	1.0
56	14<br>1.01E+07	14<br>6.47E+06	10<br>5.71E+06	12<br>7.08E+06	triosephosphate isomerase	27432.4	4.8	AIA27352.1	38.3	134.92	1.0	-1.4	-1.1
57	17<br>1.11E+07	11<br>1.87E+06	8<br>2.16E+06	21<br>9.78E+06	2-amino-3-ketobutyrate CoA ligase	43020	5.2	KMS24820.1	24.8	130.7	-1.5	-2.1	1.2
58	23	33	41	43		31769.9	5.47	AIA27732.1	33.1	130.31			

	6.50E+07	4.23E+07	2.35E+07	9.90E+07	succinyl-CoA synthetase subunit alpha						+1.4	+1.7	+1.8
59	76<br>4.65E+07	45<br>2.85E+07	20<br>1.09E+07	53<br>3.74E+07	glyceraldehyde-3-phosphate dehydrogenase	37149.7	5.95	AIA28213.1	31.3	128.92	-1.6	-3.8	-1.4
60	14<br>3.91E+06	10<br>9.63E+05	10<br>1.25E+06	11<br>1.24E+06	hypothetical protein EX97_08030	35180.9	5.6	AIA28113.1	31	128.32	-1.4	-1.4	-1.2
61	14<br>5.68E+06	7<br>7.75E+05	3<br>2.03E+05	4<br>4.90E+05	molecular chaperone GroEL	57629.2	4.55	AIA28527.1	25.2	126.76	-2.0	-4.6	-3.5
62	9<br>1.16E+07	18<br>1.02E+07	7<br>3.77E+06	17<br>1.47E+07	ferritin	19645.3	4.67	AIA28414.1	40.3	126.41	+2.0	-1.2	+1.8
63	6<br>4.56E+06	11<br>3.10E+06	7<br>9.59E+05	10<br>4.93E+06	GMP synthase	58514.6	5.03	AIA26976.1	21.8	123.55	+1.8	+1.1	+1.6
64	20<br>1.20E+07	14<br>3.36E+06	11<br>1.54E+06	18<br>8.39E+06	FAA hydrolase family protein	33197.8	4.84	KMR76054.1	34.3	122.29	-1.4	-1.8	-1.1
65	16<br>1.33E+07	9<br>4.40E+06	7<br>1.23E+06	17<br>1.08E+07	aldehyde dehydrogenase	53845.8	5.08	AHZ98099.1	20.2	121.17	-1.7	-2.2	1.0
66	14<br>1.26E+07	8<br>4.40E+06	6<br>1.21E+06	16<br>1.08E+07	aldehyde dehydrogenase	53748.7	5.12	OWU35798.1	20.2	104.92	-1.7	-2.3	1.1
67	13<br>1.00E+07	6<br>1.32E+06	5<br>1.31E+06	8<br>3.83E+06	NADH dehydrogenase	44388.8	4.66	AIA27438.1	23.3	120.69	-2.1	-2.6	-1.6
68	18<br>9.37E+06	7<br>7.15E+05	5<br>6.20E+05	10<br>2.81E+06	asparagine--tRNA ligase	49341.8	5.25	ALK41854.1	20	120.65	-2.5	-3.6	-1.8
69	13<br>2.59E+07	30<br>2.64E+07	20<br>1.37E+07	26<br>4.72E+07	alkylhydroperoxidase	21147.4	5.57	AIA26967.1	40.7	120.5	+2.3	+1.5	+2.0
70	14<br>4.68E+06	4<br>1.02E+05	6<br>8.59E+05	14<br>2.08E+06	methenyltetrahydrofolate cyclohydrolase	30900.1	5.38	AIA27555.1	47.2	120.12	-3.5	-2.3	1.0
71	12<br>3.17E+06	4<br>1.02E+05	4<br>5.10E+05	11<br>1.49E+06	methenyltetrahydrofolate cyclohydrolase	30886	5.38	AHZ98851.1	47.2	117.37	-3.0	-3.0	1.0
72	16<br>2.43E+07	17<br>8.44E+06	22<br>5.00E+05	29<br>3.35E+07	universal stress protein UspA	18531.6	5.6	AIA28237.1	39.1	116.13	1.0	+1.3	+1.8
73	21<br>2.03E+07	25<br>1.45E+07	27<br>3.16E+06	21<br>1.83E+07	peroxidase	18731.8	4.73	KMR43288.1	56.2	115.54	1.1	1.2	1.0

74	20<br>1.63E+07	12<br>5.29E+06	6<br>8.46E+05	17<br>9.85E+06	phosphoglucosamine mutase	49426.8	4.65	AQQ90729.1	19.2	115.25	-1.6	-3.3	-1.1
75	13<br>4.78E+06	7<br>1.23E+06	9<br>9.72E+05	11<br>2.33E+06	2-oxoglutarate dehydrogenase	105428.1	5.41	KMR74703.1	11.6	114.17	-1.8	-1.4	-1.1
76	9<br>5.38E+06	11<br>4.57E+06	8<br>7.51E+05	7<br>4.68E+06	DNA-directed RNA polymerase subunit alpha	35068.1	4.69	AIA28711.1	33.4	113.99	+1.2	-1.1	-1.2
77	17<br>8.90E+06	11<br>2.16E+06	10<br>1.53E+06	13<br>5.49E+06	Asp-tRNA(Asn)/Glu-tRNA(Gln) amidotransferase GatCAB subunit A	53019.1	5.02	OWU40549.1	15.8	113.74	-1.5	-1.7	-1.3
78	3<br>1.39E+06	1<br>2.79E+05	0<br>0.00E+00	2<br>1.59E+05	seryl-tRNA synthetase	48924.4	5.02	AIA26601.1	21.4	113.57	-3.0	-	-1.5
79	2<br>1.83E+06	2<br>3.09E+05	0<br>0.00E+00	2<br>4.59E+04	catalase	58505		KMS15118.1	21.9	113.01	1.0	-	1.0
80	19<br>4.45E+07	17<br>4.40E+07	11<br>2.24E+07	31<br>5.71E+07	type VII secretion protein EssB	11036.1	5.67	AIA26864.1	70.1	112.81	-1.1	-1.7	1.6
81	18<br>2.33E+07	12<br>7.74E+06	15<br>4.45E+06	15<br>2.30E+07	50S ribosomal protein L6	19786.2	9.54	AIA28722.1	46.6	112.47	-1.5	-1.2	-1.2
82	6<br>1.67E+06	4<br>1.01E+06	1<br>1.16E+04	3<br>1.27E+06	adenylosuccinate synthetase	47863.2	5.1	AIA26609.1	21.5	112.3	-1.5	-6.0	-2.0
83	8<br>2.17E+06	3<br>6.78E+05	5<br>5.08E+05	4<br>8.53E+05	universal stress protein UspA	15225.4	9.61	AIA28233.1	52.5	112.19	-2.6	-1.6	-2.0
84	14<br>1.39E+07	14<br>4.36E+06	12<br>1.82E+06	17<br>1.23E+07	inorganic pyrophosphatase	34269.4	4.69	KMR20623.1	25.2	111.6	1.0	-1.1	+1.2
85	13<br>7.04E+06	7<br>1.40E+06	5<br>7.92E+05	10<br>3.18E+06	glycyl-tRNA ligase	53904.6	4.99	AIA28105.1	16.6	110.51	-1.8	-2.6	-1.3
86	0<br>0.00E+00	0<br>0.00E+00	9<br>8.10E+05	5<br>3.17E+05	phage infection protein	47870.3	9.3	OWU48909.1	22.1	110.17	-	UP	UP
87	6<br>1.30E+06	3<br>6.87E+05	5<br>8.43E+05	4<br>1.47E+06	branched-chain amino acid aminotransferase	40085.5	4.91	AIA27111.1	32.4	109.22	-2.0	-1.2	-1.5
88	13<br>3.32E+07	26<br>1.79E+07	17<br>1.53E+07	23<br>4.04E+07	30S ribosomal protein S2	29150.9	5.44	AIA27742.1	35.2	108.11	+2.0	+1.3	+1.7
89	17	9	5	9	dihydroorotase	46901.5	5.06	AIA27687.1	21.4	107.19			

	4.74E+06	3.31E+05	5.24E+05	1.34E+06							-1.8	-3.4	-1.8
90	10<br>1.34E+07	14<br>8.89E+06	9<br>3.07E+06	10<br>9.40E+06	transaldolase	25833.1	4.72	AQQ84479.1	43.8	105.69	+1.4	-1.1	1.0
91	8<br>8.98E+06	11<br>5.66E+06	8<br>2.48E+06	11<br>1.10E+07	hypothetical protein ER16_04460	19326.4	4.87	AHZ98808.1	42	104.7	+1.3	1.0	+1.3
92	13<br>1.27E+07	15<br>7.64E+06	11<br>2.73E+06	16<br>1.56E+07	hypothetical protein EX97_04825	19325.5	5.04	AIA27510.1	38.4	101.95	+1.1	-1.1	+1.2
93	12<br>5.76E+06	8<br>1.26E+06	9<br>2.13E+06	13<br>2.44E+06	alanine dehydrogenase	40104.5	5.58	AIA28236.1	26.8	104.63	-1.5	-1.3	1.0
94	2<br>2.73E+06	2<br>1.03E+05	1<br>7.13E+04	5<br>3.68E+06	lactate dehydrogenase	34696.9	5.1	AIA26821.1	31.8	104.24	1.0	-2.0	+2.5
95	14<br>2.33E+07	9<br>7.98E+06	12<br>6.37E+06	14<br>1.72E+07	glyceraldehyde-3-phosphate dehydrogenase	36394.3	4.89	AIA27350.1	18.7	104.19	-1.5	-1.1	1.0
96	7<br>2.78E+06	3<br>6.54E+04	2<br>2.74E+05	3<br>1.90E+06	polynucleotide phosphorylase	77504.8	4.89	KMS43492.1	13.4	104.11	-2.3	-3.5	-2.5
97	12<br>2.11E+07	21<br>2.20E+07	16<br>1.21E+07	12<br>2.16E+07	50S ribosomal protein L25	23787.3	4.39	AIA27058.1	26.7	102.82	+1.7	+1.3	1.0
98	13<br>1.80E+07	14<br>7.02E+06	6<br>1.67E+06	15<br>1.14E+07	30S ribosomal protein S4	23012.9	10.02	AIA28246.1	28.5	101.96	1.0	-2.1	+1.1
99	7<br>1.55E+06	6<br>1.10E+06	2<br>1.90E+05	3<br>6.90E+05	starvation protection protein/ general stress protein	16748.4	4.57	AIA28633.1	51	101.77	-1.1	-3.5	-2.3
100	12<br>3.46E+06	7<br>6.79E+05	2<br>8.07E+04	11<br>2.87E+06	cell division protein FtsA	53104.9	4.48	AIA27671.1	14.8	100.86	-1.7	-6.0	-1.1

References:

1. Viveiros M, Rodrigues L, Martins M, Couto I, Spengler G, Martins A, Amaral L. 2010. Evaluation of efflux activity of bacteria by a semi-automated fluorometric system. Methods Mol Biol. 642:159-72. https://doi.org/10.1007/978-1-60327-279-7_12.

2. Kumar S, Rai AK, Mishra MN, Shukla M, Singh PK, Tripathi AK. 2012. RpoH2 sigma factor controls the photooxidative stress response in a non-photosynthetic rhizobacterium, *Azospirillum brasilense* Sp7. Microbiology 158: 2891-2902. https://doi.org/10.1099/mic.0.062380-0.
